# Investigating the Intercellular Spreading Properties of the Foamy Virus Gag Protein

**DOI:** 10.1371/journal.pone.0031108

**Published:** 2012-02-29

**Authors:** Joelle Tobaly-Tapiero, Alessia Zamborlini, Patricia Bittoun, Ali Saïb

**Affiliations:** 1 Institut Universitaire d'Hématologie, CNRS UMR7212-Inserm U944-Université Paris Diderot-Paris7, Paris, France; 2 Conservatoire National des Arts et Métiers, Paris, France; Queensland Institute of Medical Research, Australia

## Abstract

Small regions called protein transduction domains (PTDs), identified in cellular and viral proteins, have been reported to efficiently cross biological membranes. Here we show that the structural Gag protein of the prototypic foamy virus (PFV) is apparently able to move from cell to cell and to transport the green fluorescent protein (GFP) from few transfected cells to the nuclei of the entire monolayer. Deletion studies showed that this property lies within the second glycine/arginine (GRII) box in the C-terminus of the protein. We also found that uptake and nuclear accumulation of Gag GRII expressed as GFP-fusion protein in recipient cells was observed only following methanol fixation, but never in living cells or when cells were fixed with glutaraldehyde or treated with trichloroacetic acid prior to methanol fixation. Absence of intercellular spreading *in vivo* was further confirmed using a sensitive luciferase activity assay based on transactivation of the PFV long terminal repeats. Thus, we conclude that intercellular spreading of PFV Gag represents an artificial diffusion event occurring during cell fixation, followed by nuclear retention mediated by the chromatin-binding sequence within the Gag GRII box. In light of these results, we advise caution before defining a peptide as PTD on the basis of intercellular spreading observed by fluorescence microscopy.

## Introduction

Foamy viruses (FVs) are complex animal retroviruses, which encode for structural (Gag, Env), enzymatic (Pol) and regulatory proteins such as the transactivator protein, Tas. FVs differ in many aspects of their replication from orthoretroviruses, such as the human immunodeficiency viruses (HIV) (for a review [1]). Among other peculiarities, the prototypic foamy virus (PFV) Gag precursor lacks the landmarks of orthoretrovirus Gag polyproteins, such as the major homology region and the Cys-His motif, and rather displays unique distinctive features. The N-terminus of PFV Gag harbors a coiled-coil motif that interacts with the light chain 8 of cellular dynein, allowing microtubule-dependent trafficking of incoming viral particles towards the microtubule organizing center [2]. Three short glycine-arginine-rich (GR) boxes were mapped in the C-terminus of PFV Gag and have been implicated in viral genomic RNA packaging, reverse transcription and viral particles morphogenesis. In particular, the GRI domain binds to viral nucleic acids *in vitro* [3], while the GRII box contains an essential chromatin-binding sequence (CBS) that, by interacting with histone proteins, allows tethering of incoming PFV pre-integration complex to host chromosomes prior to viral genome integration [4]. The GRII box was described as harboring also a nuclear localization sequence (NLS) [5], a finding recently challenged [6]. Finally, the GRIII box can functionally complement the GRI box and vice versa [7].

Protein transduction domains (PTDs) are short basic-rich domains able to penetrate in nearly every cell type by directly crossing the plasma membrane. Since they also allow the simultaneous uptake of a wide array of conjugated cell-impermeable biomolecules (polypeptides, polynucleotides, small molecule drugs, 40-nm iron nanoparticles,…), PTDs have emerged as powerful tools for the manipulation of cellular functions at the protein level with a wide range of potential applications in biological and medical research [8], [9]. PTDs are found in proteins of either cellular or viral origin such as the *Drosophila* homeobox Antennapedia protein [10], the human immunodeficiency virus type 1 transcriptional factor Tat [11], [12] and herpes simplex virus type 1 (HSV-1) tegument protein VP22 [13]. Synthetic homopolymers consisting of 6–8 basic residues (arginine, lysine or ornitine) also endow with cell-penetrating properties [14]. Initial studies reported that uptake is rapid and receptor- and energy-independent [15]. These observations have subsequently been attributed to accumulation of PTDs on the cell surface via electrostatic interaction with negatively-charged molecules and/or artefactual redistribution of internalized PTDs following fixation [16], [17], [18], [19]. Several recent reports suggested that uptake might rely on both endocytic and nonendocytic mechanisms, depending on several parameters such as the concentration and the physiochemical properties of the PTD and/or the associated cargo [20]. However, the internalization mode of PTDs that would grant access to target sites in the cytoplasm and/or the nucleus, thus yielding the biological effects, still remains unclear and a matter of controversies.

Here, we report that the PFV Gag protein exhibits the ability to spread from actively synthesizing cells to surrounding ones. By analyzing the transcellular movement of Gag C-terminal truncation mutants, we mapped this property to the GRII box. The intercellular trafficking of the green fluorescent protein (GFP) fused to either PFV Gag GRII box (GFP-Gag_GRII_) or the C-terminal domain of VP22 (GFP-VP22_Cter_), which harbors a previously characterized PTD [13], was observed upon fixation with methanol, but never in living cells or when cells were fixed with glutaraldehyde, or treated with trichloroacetic acid (TCA) known to precipitate soluble proteins prior to methanol fixation. We also established that, when GFP-Gag_GRII_ or GFP-VP22_Cter_-expressing cells were fixed with methanol in close contact to mock-transfected ones, the latter became GFP-positive. Finally, we show that expression of Gag GRII and full-length VP22, as fusion proteins with the PFV Tas protein, in cells harboring the luciferase gene under the control of the PFV LTR, induced luciferase activity. On the contrary, transactivation was not detected when cells expressing either Gag_GRII_-Tas or VP22-Tas were co-cultured with those harboring a plasmid encoding the PFV LTR, further supporting the absence of intercellular spreading *in vivo*. We conclude that uptake and nuclear accumulation of PFV Gag in naïve recipient cells represents an artificial diffusion event occurring during cell fixation with subsequent nuclear retention mediated by the CBS within the GRII box [4], [7].

## Results

**Figure 1:**
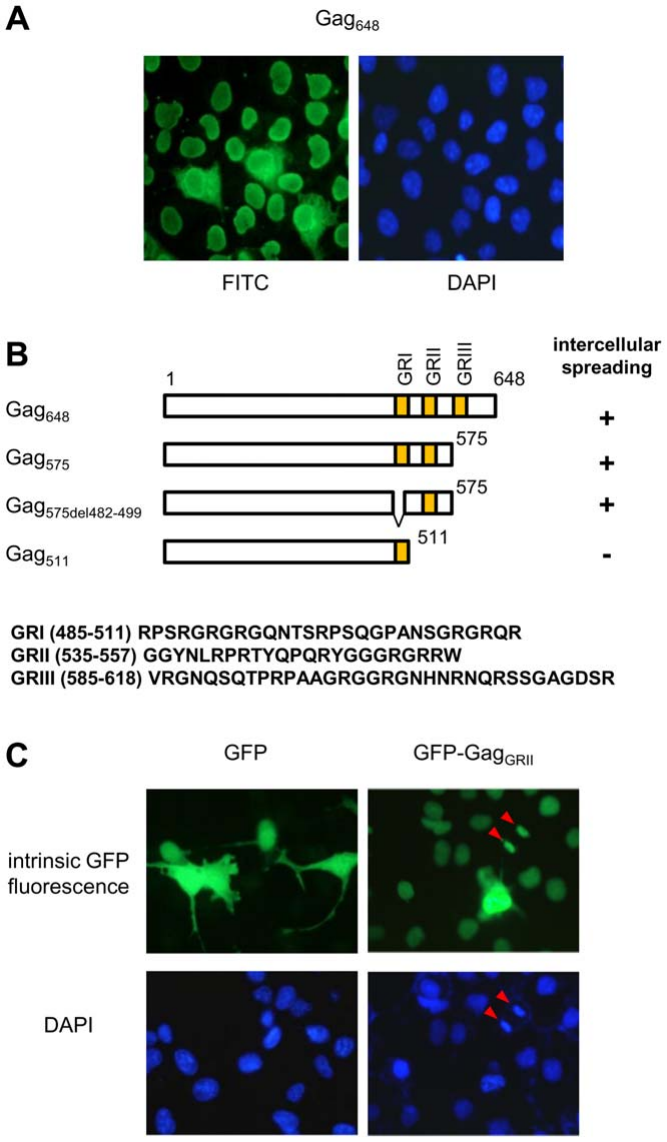
Intercellular spreading of full-length and C-terminal deletion mutant PFV Gag proteins. **A**. Distribution of full-length Gag (Gag_648_) in cells was analyzed by indirect immunofluorescence. Forty-eight hours after transfection, cells were fixed with methanol, labeled with anti-Gag antibodies followed by fluorescein-isothiocyanate coupled secondary antibodies and visualized on a fluorescence microscope. Nuclei were stained with DAPI. Original magnification ×40. **B**. The intercellular trafficking of full-length and C-terminal deletion mutant Gag proteins was followed as in A. In the illustration, numbers mark the first and last amino acids which are preserved after deletion. The glycin/arginine-(GR)-rich boxes are indicated by shaded regions and their location and amino acids composition are represented below according to [31]. The plus sign corresponds to 100% of positive cells, whereas the minus sign indicates that only transfected cells are positive. **C**. Following methanol fixation, cells expressing GFP or GFP-Gag_GRII_ were examined for intrinsic fluorescence. Nuclei are stained with DAPI. Original magnification ×40. Red arrows indicate a cell undergoing mitosis.

PFV Gag displays different patterns of subcellular distribution, being localized in the cytoplasm, the nucleus and the centrosome in transfected and infected cells [5], [21], [22]. We also noticed that, 48 hours post-transfection of Cos6 cells with a full-length Gag-expressing vector (Gag648), the entire cell monolayer stained positive in an indirect immunofluorescence assay using anti-Gag antibodies, while no signal was observed upon incubation of mock-transfected cells. Intense Gag staining was detected both in the cytoplasm and the nucleus in some cells that likely represent the transfected ones, while it was strictly confined in the nucleus in the rest of the cell culture (Fig. 1A). Similar observations were made upon PFV Gag expression in other cell types, such as hamster BHK21 or human U373MG cells (data not shown). By studying the distribution of C-terminal truncated forms of Gag by indirect immunofluorescence, we also found that the staining pattern of Gag mutants lacking the GRIII box alone (Gag_575_) or together with the GRI box (Gag_575del482–499_) was similar to that of the wild-type counterpart, with few intensely fluorescent cells surrounded by numerous cells harboring nuclear staining (Fig. 1B). In contrast, the expression of the Gag mutant missing both the GRII and GRIII domains (Gag_511_) was restricted only to some cells (fig. 1B). These results suggest that the PFV Gag region harboring the GRII box is responsible for the intercellular trafficking of the protein.

Spreading from actively synthesizing cells to neighboring ones is a property attributed to PTD-carrying proteins. We therefore asked whether PFV Gag harbors a PTD within the GRII box. To answer this question, we expressed the GRII box fused to GFP (GFP-Gag_GRII_) into Cos6 cells, which were fixed with methanol 48 h later and analyzed for intrinsic fluorescence by microscopy. While the parental GFP remained confined to expressing cells, we found that the 23 amino acids peptide, spanning from aa 535 to aa 557 of PFV Gag, was sufficient to allow the extension of the GFP signal from some transfected cells to the nucleus of surrounding ones (Fig. 1C). We noticed also that the GFP-fusion protein painted condensed chromosomes in cells undergoing mitosis (Fig. 1C, red arrows), consistent with our previous findings that the GRII box harbors a CBS [4].

As previously reported for other PTDs [23], intercellular trafficking of GFP-Gag_GRII_ still occurred following treatment with Brefeldin A (BFA), a reversible inhibitor of Golgi-mediated protein secretion, or incubation at 4°C known to block endo/exocytosis prior processing for fluorescence microscopy, or upon GFP-Gag_GRII_ expression in gap junction-negative Neuro2A cells (data not shown). Altogether these findings indicate that the GRII box of PFV Gag displays the features of a PTD.

**Figure 2:**
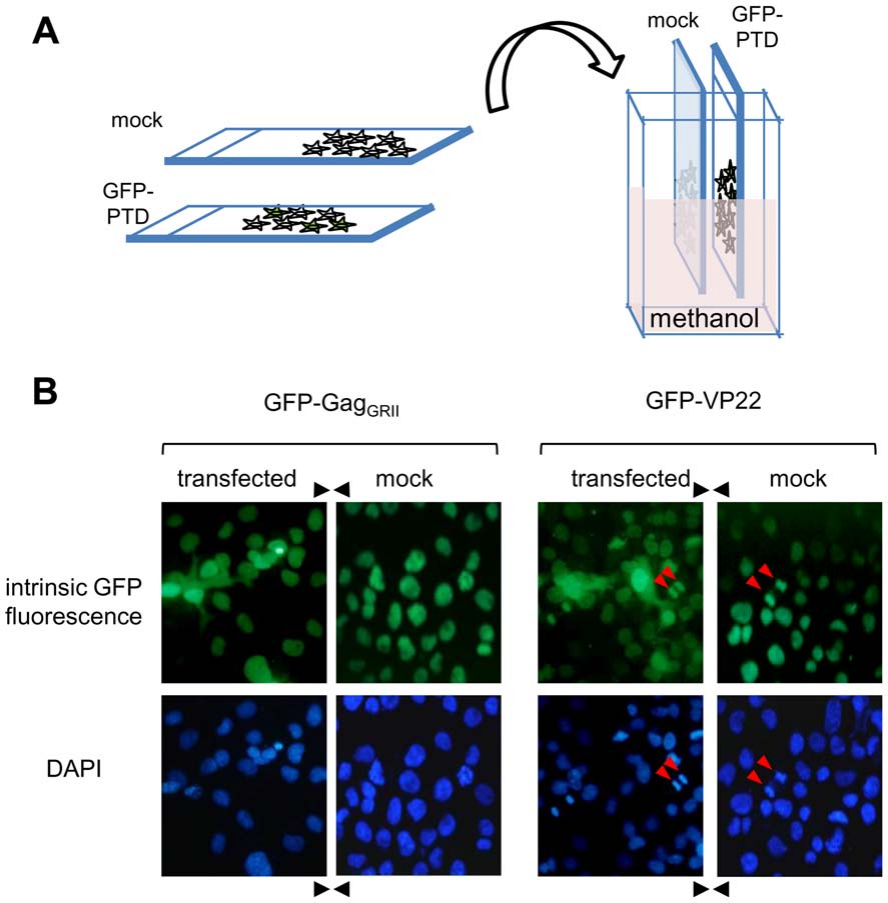
Extension of GFP fluorescence from cells expressing GFP-Gag_GRII_ or GFP-VP22_Cter_ to recipient ones during fixation. **A**. Representation of the experimental procedure followed: a slide coated with cells expressing GFP-Gag_GRII_ or GFP-VP22_Cter_ was abutted head to head to a slide of mock-transfected cells and, then, fixed in close contact. **B**. GFP-Gag_GRII_- or GFP-VP22-expressing cells (transfected) and mock-transfected cells (mock) were fixed with methanol in close contact and next visualized on a fluorescence microscope. Original magnification ×40. Red arrows indicate a cell undergoing mitosis.

Although intercellular spreading of GFP-Gag_GRII_ was readily visualized in methanol-fixed cells, we were unable to observe GFP signal in the nucleus of recipient live cells (data not shown). Similar results were previously reported for HSV-1 VP22 protein fused to GFP [18], [24], [25], [26], [27]. In that case, failure to detect direct fluorescence in recipient cells was initially attributed to levels of the GFP-fusion protein below the detection limit or to unfolding and/or quenching of GFP, which could be reverted upon methanol fixation and rehydratation. However, subsequent studies indicated that the internalization and nuclear accumulation of VP22 observed *in vitro* might be artificial, resulting from protein leakage upon cell fixation ([28]. To define whether translocation of GFP-Gag_GRII_ from expressing cells to the nucleus of neighboring ones is authentic or just a result of protein extraction during the fixation step, we reproduced a previously designed experiment in which a slide coated with cells expressing GFP fused to either PFV Gag_GRII_ or the C-terminal region of VP22 (aa 100–301), which harbors the PTD [13], was abutted head to head to a slide of mock-transfected cells (Fig. 2A). The two slides were then fixed in close contact with methanol and, next, examined by fluorescence microscopy. As expected, 100% of GFP-Gag_GRII_ or GFP-VP22_Cter_-transfected cells were found positive for GFP (Fig. 2B). The entire mock-transfected monolayer fixed in proximity of GFP-VP22_Cter_-expressing cells stained GFP-positive (Fig. 2B), as already reported for wild-type VP22 [24]. More importantly, the same observation was made in the case of the mock-transfected slide fixed in contact with cells expressing GFP-Gag_GRII_ (Fig. 2B).

**Figure 3:**
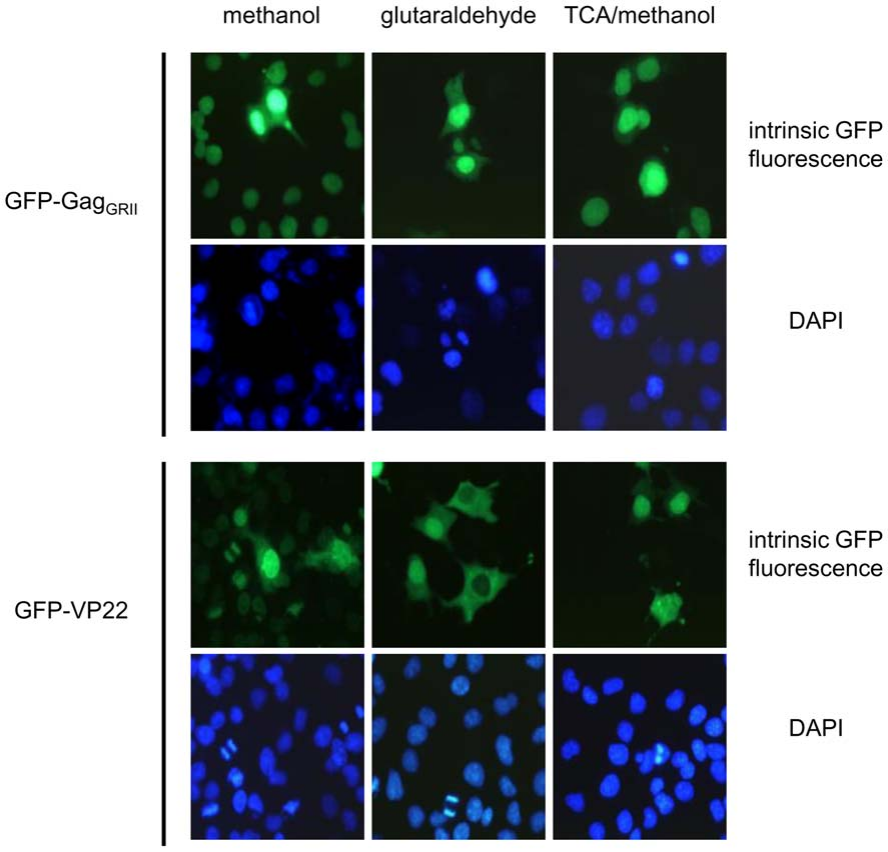
The fixation procedure influences the intercellular spreading of GFP-Gag_GRII_ and GFP-VP22. Cells expressing GFP-Gag_GRII_ or GFP-VP22 were fixed with methanol, glutaraldehyde or treated with TCA before fixation with methanol, as indicated. Cells were then analyzed for intrinsic fluorescence. Original magnification ×40.

While fixing cellular structures, cold organic solvents (e.g. methanol) concomitantly induce membrane permeabilization and might therefore trigger differential extraction or redistribution of soluble proteins or of proteins weakly or transiently associated with cellular membranes, leading to an artificial localization. To investigate further the intercellular trafficking properties of GFP-Gag_GRII_ and GFP-VP22_Cter_, we compared their subcellular localization after fixation of the transfected cell monolayer with methanol, which fixes proteins by dehydration and precipitation, or glutaraldehyde, which forms a stable covalent network through intermolecular bridges between proteins [29]. In agreement with published data [24], and with the results presented above (Fig. 1C), when cells expressing GFP-Gag_GRII_ or GFP-VP22_Cter_ were fixed with methanol, secondary recipient cells displayed evident nuclear fluorescence (Fig. 3, left panel). On the contrary, following treatment with glutaraldehyde only a small number of GFP-positive cells could be observed in either case (Fig. 3, middle panel). Finally, to provide direct evidence that leaking occurs during fixation, we reasoned that treatment of transfected cells with TCA, which causes the quantitative precipitation of soluble proteins, prior to methanol fixation, should not affect the trafficking of proteins harboring genuine intercellular spreading properties. As shown in figure 3 (right panel), TCA treatment prevented the extension of the fluorescence to cells surrounding those expressing either GFP-Gag_GRII_ or GFP-VP22_Cter_, without affecting the intrinsic GFP signal. These results demonstrate that protein precipitation before methanol fixation efficiently blocked intercellular spreading of both GFP-fusion proteins and support the hypothesis that extension of GFP fluorescence to recipient cells adjacent to actively synthesizing ones is due to leakage during the fixation procedure.

Failure to observe intercellular spreading of GFP-fusion proteins might result from quenching of intrinsic fluorescence by improper folding of GFP and/or by low protein levels. To overcome these limitations we assessed such properties using a sensitive reporter gene transcription assay based on the transactivation of the PFV LTR by the viral Tas protein. We therefore generated vectors encoding Gag GRII or full-length VP22, which harbors a previously characterized PTD [13] and was used here as positive control, fused in frame to the N-terminus of Tas. Next, Gag_GRII_-Tas, VP22-Tas or wild-type Tas were used to transfect Cos6 cells together with a plasmid encoding for the *firefly* luciferase gene under the control of the PVF LTR (pLTR-Luc) or the corresponding promotor-less counterpart (pGL3 basic). A plasmid harboring the *renilla* luciferase driven by the cytomegalovirus immediate early promoter (pRL-CMV) was also co-transfected and used for normalization. Forty-eight hours later, transactivation of the viral LTR was determined by measuring the luciferase activity. Either Gag_GRII_-Tas or VP22-Tas was able to induce reporter gene expression, despite at a lower extent than wild-type Tas, suggesting that the secondary structure of the fusion proteins might influence their function (Table 1, direct). However, the relative fold induction compared to the background signal remained significant (Table 1). We next assessed whether the Tas-fusion proteins could undergo intercellular transport by co-culture experiments. Twenty-four hours after transfection, Cos6 cells expressing wild-type Tas or Gag_GRII_-Tas or VP22-Tas were mixed with cells bearing both the pLTR-Luc and the pRL-CMV plasmids (ratio 1∶1) and the luciferase activity was measured 48 h later. None of these proteins was able to transactivate the PFV LTR under these settings (Table 1, indirect). Changes in the experimental conditions (time post-transfection, ratio of transfected cells and time of co-culture) never lead to LTR transactivation. These results further support the absence of intercellular spreading of PFV Gag GRII and VP22 *in vivo*.

**Table 1:** Lucifarase activity following transactivation of the PFV LTR.

	Fold increase		
constructs	WT Tas	GRII-Tas	VP22-Tas
pGL3basic	0.5	1	1
LTR-Luc direct	45	13	8
LTR-Luc indirect	1	1	0.1

Activation of the luciferase reporter gene was measured following expression of wild-type Tas or Gag_GRII_-Tas or VP22-Tas in Cos6 cells harboring the pLTR-Luc/pRL-CMV plasmids (direct). The normalized luciferase activity measured in cells transfected with the appropriate empty vector was set to 1. Alternatively, cells expressing wild-type Tas or Gag_GRII_-Tas or VP22-Tas were co-cultured with recipient cells harboring the pLTR-Luc/pRL-CMV plasmids (indirect). Values are representative of three independent transfection experiments performed in duplicate.

## Discussion

In the past decade, protein transduction has emerged as a promising approach for the direct intracellular delivery of a wide array of biologically relevant molecules in applications ranging from basic research on cell functions to the development of therapeutics for the treatment of human diseases [8], [9]. Despite the field suffered from the controversies about the efficacy of PTD-mediated delivery [16], [17], [18], [19] and several drawbacks such as poor specificity and endosomal entrapment of PTD-cargo are unsolved, the promise of high yield of delivery, low toxicity, versatility of cargo and wide range of target cell types still stimulates the search for novel peptides with transduction properties [30].

During our studies on PFV Gag trafficking, we observed that it displays transcellular movement [31] and decided to investigate this property further. Here, we show that intercellular spreading of Gag can be attributed to the GRII box. Upon fixation with methanol of a cell monolayer transfected with a plasmid encoding for GFP-Gag_GRII_, we found that virtually every cell exhibited a bright nuclear fluorescence, while spreading was not observed in live cells. Similar results have been reported for HSV-1 VP22, which uptake has already been suggested to be a post-fixation artifact [28]. In that case, the protein was shown to adhere to the cell surface, likely via its PTD, and to penetrate in the cytoplasm and/or the nucleus following loss of membrane integrity upon fixation [19], [28]. Persistent accumulation in the nucleus following import was ascribed to the affinity of VP22 for chromatin [32], [33], [34]. Likewise, the PFV Gag GRII box harbors a CBS that mediates tethering to cell chromosomes [4], explaining its enrichment in the nucleus of secondary recipient cells. Of note, we consistently observed that GFP fused to either Gag_GRII_ or VP22 painted mitotic chromosomes (Fig. 1C and 2B).

Fixation prior to microscopy studies is critical to preserve cellular structures and proteins distribution. Thus an inappropriate procedure might yield factitious observations due to redistribution or leakage of soluble protein from permeabilized cells [35]. Noteworthy, while control experiments typically address the specificity of the antibodies, little care is frequently paid on the choice of the fixation agent, which is often based on routine practice rather than on systematic comparison of different methods. By using different fixation procedures, we established that spreading and nuclear localization of the PFV Gag is due to artificial transport occurring during cell fixation, as already observed in the case of VP22 [19], [28]. More importantly, by showing that fluorescence is strictly confined to PFV Gag_GRII_- or HSV-1 VP22-expressing cells when soluble proteins are precipitated by TCA treatment prior to methanol fixation in order to preserve faithful localization prior to membrane permeabilization, our results indicate that intercellular spreading is due to post-fixation extraction of soluble proteins. This conclusion is further supported by the finding that expression of Gag_GRII_ or full-length VP22 as fusion protein with Tas is not sufficient to allow the delivery of the viral transactivator into co-cultured cells harboring the luciferase gene driven by the PFV LTR, as indicated by absence of reporter gene expression.

Although it cannot be excluded that a small amount of protein might translocate across the cell membrane, and/or that a cargo fused to the PTD might affect its structure thus interfering with the delivery of the complex, several studies including the present strongly point out that caution should be used before defining a novel PTD. Additionally, it is currently clear that fluorescence microscopy studies on fixed cells, an accessible and widely used technique to determine the subcellular distribution of a protein, is not a technique suited to address the genuine transcellular properties of such domains.

## Materials and Methods

### Plasmid constructions

Gag expression plasmids, corresponding to the entire Gag ORF (Gag648) and C-terminal deletion mutants were constructed as previously described [31]. To produce the GFP-Gag_GRII_ expressing vector, we generated a duplex encoding for the GRII box (aa 535–557 of PFV Gag) flanked by cohesive ends by annealing of specific complementary oligonucleotides. The annealing reaction mixture (50 µl) containing 20 µl of each oligonucleotide (50 µM), 25 mM Tris-HCl pH 8, 10 mM MgCl_2_, was heated at 95°C for 5 min and then cooled to room temperature. Five microliters of this mix were used for ligation with 200 ng of pEGFP-C1 digested with EcoRI-BamHI. GFP-VP22_C-ter_ containing the C-terminal region of VP22 (aa 100 to 301) was obtained by inserting the BspEI-BamHI fragment of pVP22/Myc-His (Invitrogen) into pEGFP-C1. To generate a Tas-fusion protein expressing vector (pSV-Tasbx), the BglII and XhoI restriction sites were inserted in the 5′ end of the *tas* ORF into the pSV-Tas plasmid [36] using the QuickChange mutagenesis kit (Stratagene). A double strand oligonucleotide encoding for the GRII box with an ATG initiator codon and flanked by cohesive ends, was generated by annealing of single-stranded complementary of nucleotides as described above and, next, introduced into pSV-Tasbx, yielding the Gag_GRII_-Tas vector. The VP22-Tas plasmid was generated upon insertion of a PCR product encoding the entire VP22 ORF into pSV-Tasbx. To generate the pLTR-Luc vector, the PFV LTR sequence was extracted from pHFV13, which encodes the entire proviral genome of HFV, with *Bam*HI and *Hin*dIII and was inserted in the promoterless pGL3basic vector upstream of the *firefly* luciferase gene (Promega). The identity of recombinant clones was verified by DNA sequencing.

### Cell culture

Cells were grown in Dulbecco's modified Eagle medium supplemented with 5% fetal calf serum, 2 mM L-glutamine, 25 mM Hepes and antibiotics (penicillin/streptomycin), at 37°C, 5% CO2. Cos6, a SV40-transformed African green monkey kidney cell line, was purchased from ATCC.

### Transient transfection and luciferase activity assay

Cells (10E5 cells per well) were seeded into 6-well plates. Transfections were performed using Lipofectin (Gibco-BRL) with 1 µg of DNA plasmid, as specified by the manufacturer.

For the transactivation experiments, Cos6 cells were co-transfected with the pGL3basic or pLTR-Luc and pRL-CMV, which encodes the *renilla* luciferase under the control of the cytomegalovirus immediate-early promoter and is used for normalization, together with vectors encoding PFV Gag_GRII_-Tas or VP22-Tas or wild-type Tas. Alternatively, cells harboring pLTR-Luc and pRL-CMV were mixed with those expressing PFV Gag_GRII_-Tas or VP22-Tas or wild-type Tas (ration 1∶1), 24 h after transfection. The appropriate empty vector was used as negative control. After 48 h, cell supernatants were analyzed for luciferase activity in duplicate using the Dual-Luciferase Reporter Assay System (Promega), an EG&G Berthold Microplate LB 96 V luminometer, and a Microlite 1 flat-bottom microtiter plate (Thermo Labsystems).

### Fluorescence microscopy

Forty-eight hours after transfection, cells were washed twice with PBS and were prepared for monitoring the GFP fluorescence according to different treatment conditions: (i) Cells were fixed with 100% methanol at room temperature (RT) for 15 min and then rinsed with PBS. (ii) Cells were fixed with 1% glutaraldehyde 1 h at RT. (iii) Cells were pretreated with 5% tri-chloro-acetic acid (TCA) overnight at 4°C before methanol. For indirect immunofluorescence, Gag proteins were detected with rabbit polyclonal anti-Gag antiserum and revealed with secondary fluorescein isothiocyanate-coupled antibodies. Following incubation with 4′,6-diamidino-2-phenylindole (DAPI) (250 ng/ml) to stain the nucleus, cells were embedded in Mowiol (Calbiochem) and were examined with a Bio-Rad MRC-1024 confocal imaging system (Hertforshire, United Kingdom) and an inverted Diaphot 300 Nikon microscope. Images were collected with an oil immersion objective (40×, NA I0.4 plan Apochromat).

## References

[1] Delelis O, Lehmann-Che J, Saib A (2004). Foamy viruses - a world apart.. Curr Opin Microbiol.

[2] Petit C, Giron ML, Tobaly-Tapiero J, Bittoun P, Real E (2003). Targeting of incoming retroviral Gag to the centrosome involves a direct interaction with the dynein light chain 8.. J Cell Sci.

[3] Yu SF, Edelmann K, Strong RK, Moebes A, Rethwilm A (1996). The carboxyl terminus of the human foamy virus Gag protein contains separable nucleic acid binding and nuclear transport domains.. J Virol.

[4] Tobaly-Tapiero J, Bittoun P, Lehmann-Che J, Delelis O, Giron ML (2008). Chromatin tethering of incoming foamy virus by the structural Gag protein.. Traffic.

[5] Schliephake AW, Rethwilm A (1994). Nuclear localization of foamy virus Gag precursor protein.. J Virol.

[6] Mullers E, Stirnnagel K, Kaulfuss S, Lindemann D (2011). Prototype Foamy Virus Gag Nuclear Localization: a Novel Pathway among Retroviruses.. J Virol.

[7] Mullers E, Uhlig T, Stirnnagel K, Fiebig U, Zentgraf H (2011). Novel functions of prototype foamy virus Gag glycine- arginine-rich boxes in reverse transcription and particle morphogenesis.. J Virol.

[8] Fonseca SB, Pereira MP, Kelley SO (2009). Recent advances in the use of cell-penetrating peptides for medical and biological applications.. Adv Drug Deliv Rev.

[9] Heitz F, Morris MC, Divita G (2009). Twenty years of cell-penetrating peptides: from molecular mechanisms to therapeutics.. Br J Pharmacol.

[10] Derossi D, Joliot AH, Chassaing G, Prochiantz A (1994). The third helix of the Antennapedia homeodomain translocates through biological membranes.. J Biol Chem.

[11] Green M, Loewenstein PM (1988). Autonomous functional domains of chemically synthesized human immunodeficiency virus tat trans-activator protein.. Cell.

[12] Frankel AD, Pabo CO (1988). Cellular uptake of the tat protein from human immunodeficiency virus.. Cell.

[13] Elliott G, O'Hare P (1997). Intercellular trafficking and protein delivery by a herpesvirus structural protein.. Cell.

[14] Jarver P, Langel U (2004). The use of cell-penetrating peptides as a tool for gene regulation.. Drug Discov Today.

[15] Schwarze SR, Hruska KA, Dowdy SF (2000). Protein transduction: unrestricted delivery into all cells?. Trends Cell Biol.

[16] Richard JP, Melikov K, Vives E, Ramos C, Verbeure B (2003). Cell-penetrating peptides. A reevaluation of the mechanism of cellular uptake.. J Biol Chem.

[17] Richard JP, Melikov K, Brooks H, Prevot P, Lebleu B (2005). Cellular uptake of unconjugated TAT peptide involves clathrin-dependent endocytosis and heparan sulfate receptors.. J Biol Chem.

[18] Elliott G, O'Hare P (1999). Intercellular trafficking of VP22-GFP fusion proteins.. Gene Ther.

[19] Lundberg M, Johansson M (2001). Is VP22 nuclear homing an artifact?. Nat Biotechnol.

[20] Duchardt F, Fotin-Mleczek M, Schwarz H, Fischer R, Brock R (2007). A comprehensive model for the cellular uptake of cationic cell-penetrating peptides.. Traffic.

[21] Saib A, Puvion-Dutilleul F, Schmid M, Peries J, de The H (1997). Nuclear targeting of incoming human foamy virus Gag proteins involves a centriolar step.. J Virol.

[22] Yu SF, Eastman SW, Linial ML (2006). Foamy virus capsid assembly occurs at a pericentriolar region through a cytoplasmic targeting/retention signal in Gag.. Traffic.

[23] Schwarze SR, Dowdy SF (2000). In vivo protein transduction: intracellular delivery of biologically active proteins, compounds and DNA.. TIPS.

[24] Brewis N, Phelan A, Webb J, Drew J, Elliott G (2000). Evaluation of VP22 spread in tissue culture.. J Virol.

[25] Elliott G, O'Hare P (1999). Live-cell analysis of a green fluorescent protein-tagged herpes simplex virus infection.. J Virol.

[26] Elliott G, O'Hare P (2000). Cytoplasm-to-nucleus translocation of a herpesvirus tegument protein during cell division.. J Virol.

[27] Fang B, Xu B, Koch P, Roth JA (1998). Intercellular trafficking of VP22-GFP fusion proteins is not observed in cultured mammalian cells.. Gene Ther.

[28] Lundberg M, Wikstrom S, Johansson M (2003). Cell surface adherence and endocytosis of protein transduction domains.. Mol Ther.

[29] Kiernan JA (2000). Formaldehyde, formalin, paraformaldehyde and glutaraldehyde: What they are and what they do..

[30] Duchardt F, Ruttekolk IR, Verdurmen WP, Lortat-Jacob H, Burck J (2009). A cell-penetrating peptide derived from human lactoferrin with conformation-dependent uptake efficiency.. J Biol Chem.

[31] Tobaly-Tapiero J, Bittoun P, Giron ML, Neves M, Koken M (2001). Human foamy virus capsid formation requires an interaction domain in the N terminus of Gag.. J Virol.

[32] Ingvarsdottir K, Blaho JA (2010). Association of the herpes simplex virus major tegument structural protein VP22 with chromatin.. Biochim Biophys Acta.

[33] Lopez MR, Schlegel EF, Wintersteller S, Blaho JA (2008). The major tegument structural protein VP22 targets areas of dispersed nucleolin and marginalized chromatin during productive herpes simplex virus 1 infection.. Virus Res.

[34] Lundberg M, Johansson M (2002). Positively charged DNA-binding proteins cause apparent cell membrane translocation.. Biochem Biophys Res Commun.

[35] Melan MA, Sluder G (1992). Redistribution and differential extraction of soluble proteins in permeabilized cultured cells.. J Cell Sci.

[36] Lecellier CH, Neves M, Giron ML, Tobaly-Tapiero J, Saib A (2002). Further characterization of equine foamy virus reveals unusual features among the foamy viruses.. J Virol.

